# Spleen granulopoiesis in psoriasis immune microenvironment aggravates psoriasis via IL-6/P-STAT3 signaling

**DOI:** 10.1186/s13062-025-00675-2

**Published:** 2025-09-02

**Authors:** Feng Shi, Pixia Gong, Shan Huang, Weidong Zhu, Chenxi Shi, Chang Qi, Zhe Lei, Yayun Ding

**Affiliations:** 1 Department of Dermatology and Venereology, The Affiliated Suzhou Hospital of Nanjing Medical University, Suzhou Municipal Hospital, Suzhou, Jiangsu 215123 China; 2 NHC Key Laboratory of Thrombosis and Hemostasis, National Clinical Research Center for Hematologic Diseases, The First Affiliated Hospital of Soochow University, Jiangsu Institute of Hematology, Suzhou, Jiangsu 215123 China; 3 Department of Pathology, The First Affiliated Hospital of Soochow University, Suzhou, Jiangsu 215123 China

**Keywords:** Psoriasis, Spleen, Neutrophil, Granulopoiesis, IL-6

## Abstract

**Background:**

Psoriasis is an immune-mediated chronic inflammatory condition characterized by significant neutrophil infiltration in the skin. Given that the spleen is the largest peripheral immune organ in the body, it is important to investigate whether it has any impact on skin inflammation in psoriasis.

**Methods:**

To investigate this mechanism, a psoriatic mouse model was established by IMQ application. Flow cytometry and immunohistochemistry analyses were performed to determine the percentage of various immune cells in the spleen. The role of neutrophils was specifically assessed using the anti-Gr-1 antibody. Splenic granulopoiesis was evaluated using EdU labeling. To understand the spleen's role in skin inflammation, splenectomy was performed on the experimental mice. IL-6 levels were measured by ELISA, and P-STAT3 in neutrophils was detected via immunofluorescence. Further examination of IL-6's effects on neutrophil formation involved treating mice with IL-6 antibody. The severity of psoriasis was evaluated through histological staining and PASI scoring.

**Results:**

Our study revealed that the spleens of psoriatic mice were enlarged compared to those of vehicle mice. Among immune cell populations, neutrophils showed the most significant changes, with marked increases in both spleen and skin of psoriatic mice and patients, contributing to disease progression. Post-splenectomy, neutrophil infiltration in the skin was reduced by approximately 60% in psoriatic mice. This indicates that the neutrophils in the skin were primarily derived from the spleen. Additionally, the spleen showed a notable capacity for granulopoiesis with elevated neutrophils. Moreover, we found elevated IL-6 levels in the skin, blood, and spleen in the model, which was decreased after splenectomy. Treatment with an IL-6 antibody reduced neutrophil formation in both the spleen and skin, which alleviated skin inflammation in psoriatic mice. Additionally, P-STAT3 signaling was decreased following IL-6 antibody treatment. The neutrophil infiltration in spleen and skin was decreased after injection with the inhibitor of P-STAT3, which also alleviated the inflammation of psoriatic model. Thus, IL-6 served as the dominant regulator of spleen granulopoiesis, a process potentially mediated by P-STAT3 signaling.

**Conclusions:**

The spleen plays a crucial role in the immune microenvironment of psoriasis as a major site of granulopoiesis, influencing neutrophil infiltration in the skin of psoriatic mice. Additionally, IL-6 is a key regulator of neutrophil formation in the spleen of psoriatic mice, likely through P-STAT3-dependent mechanisms.

**Supplementary Information:**

The online version contains supplementary material available at 10.1186/s13062-025-00675-2.

## Introduction

Psoriasis is a chronic autoimmune skin disease characterized by the presence of erythematous plaques covered with silvery scales [1]. It severely affects both the physiological and psychological well-being of individuals with psoriasis [2]. The pathogenesis of psoriasis remains a subject of onging debate. However, it is widely acknowledged that the interactions among immune cells infiltrating the lesions contribute to the excessive proliferation of keratinocyte [3].

Currently, T cell-focused therapies exhibit limited efficacy in achieving complete remission of psoriasis [4]. Emerging evidence suggests the involvement of other immune cells [5]. Neutrophils, vital innate immune components, also exert influence on adaptive responses associated with autoimmunity, inflammation, and cancer [6]. In early stages of psoriasis, neutrophils infiltrate into the epidermis [7], often coinciding with upregulated molecules like leukotriene B4 (LTB4), MM9, and CXCL1 [7, 8]. Additionally, spleen, the largest peripheral immune organ in the body, plays a critical role in various systemic diseases, with complex and diverse mechanisms. The spleen could be damaged by neutrophil metalloproteinase and then hampers the infection of trypanosomiasis [9]. Spleen-derived IL-10 plays a significant role in obesity-induced hypothalamic inflammation [10]. Thus, the spleen and peripheral tissues demonstrate a mutual regulatory relationship. However, it is not still fully understood the immune regulatory relationship between spleen and psoriasis lesions.

In the context of physiological homeostasis, hematopoietic stem cells residing in the bone marrow serve as the primary reservoir for neutrophil production [11]. However, under special pathological conditions, the spleen can transiently function as a site of hematopoiesis [12, 13]. Previous studies have reported that the spleen is capable of generating inflammatory cells that migrate to the sites of atherosclerosis, thereby hastening disease progression [14]. Additionally, in cases of myocardial infarction, noradrenalin stimulates rapid release of progenitor cells from the bone marrow. These cells then travel to the spleen where a substantial quantity of monocytes is generated, further promoting atherosclerosis advancement [15]. Furthermore, researchers conducted splenic staining in mice, followed by transplantation into recipient mice undergoing spleen extraction. Consequently, dye-labeled neutrophils originating from the spleen were detected in mouse lungs [16]. This suggests that, under specific pathological circumstances, the spleen may act as an important site for granulopoiesis outside of the bone marrow. However, whether this role extends to psoriasis remains unclear.

In our study, we observed a significant enlargement of the spleen. Notably, neutrophils were the primary cell population that exhibited a substantial increase within the spleen. Following splenectomy, a decrease in neutrophils within the lesions was noted, suggesting that the spleen may serve as the principal source of neutrophils. Additionally, we observed a significant granulopoiesis in the spleens of psoriatic mice compared to normal mice, potentially due to IL-6-mediated stimulation, which was also decreased after splenectomy. Subsequent in vivo experiments revealed a reduction in granulopoiesis upon administration of an IL-6 antibody. Additionally, both splenic and cutaneous levels of neutrophils decreased, leading to a notable attenuation of inflammation. Furthermore, P-STAT3 was decreased after IL-6 antibody treatment. Stattic, the inhibitor of P-STAT3, inhibited the neutrophil infiltration in the spleen and skin and alleviated the inflammation of psoriatic model. Consequently, our findings highlight the spleen as a central site for granulopoiesis in the IMQ-induced psoriasis model by IL-6/P-STAT3.

## Result

### The spleen significantly enlarged in the psoriatic mice

**Fig. 1:**
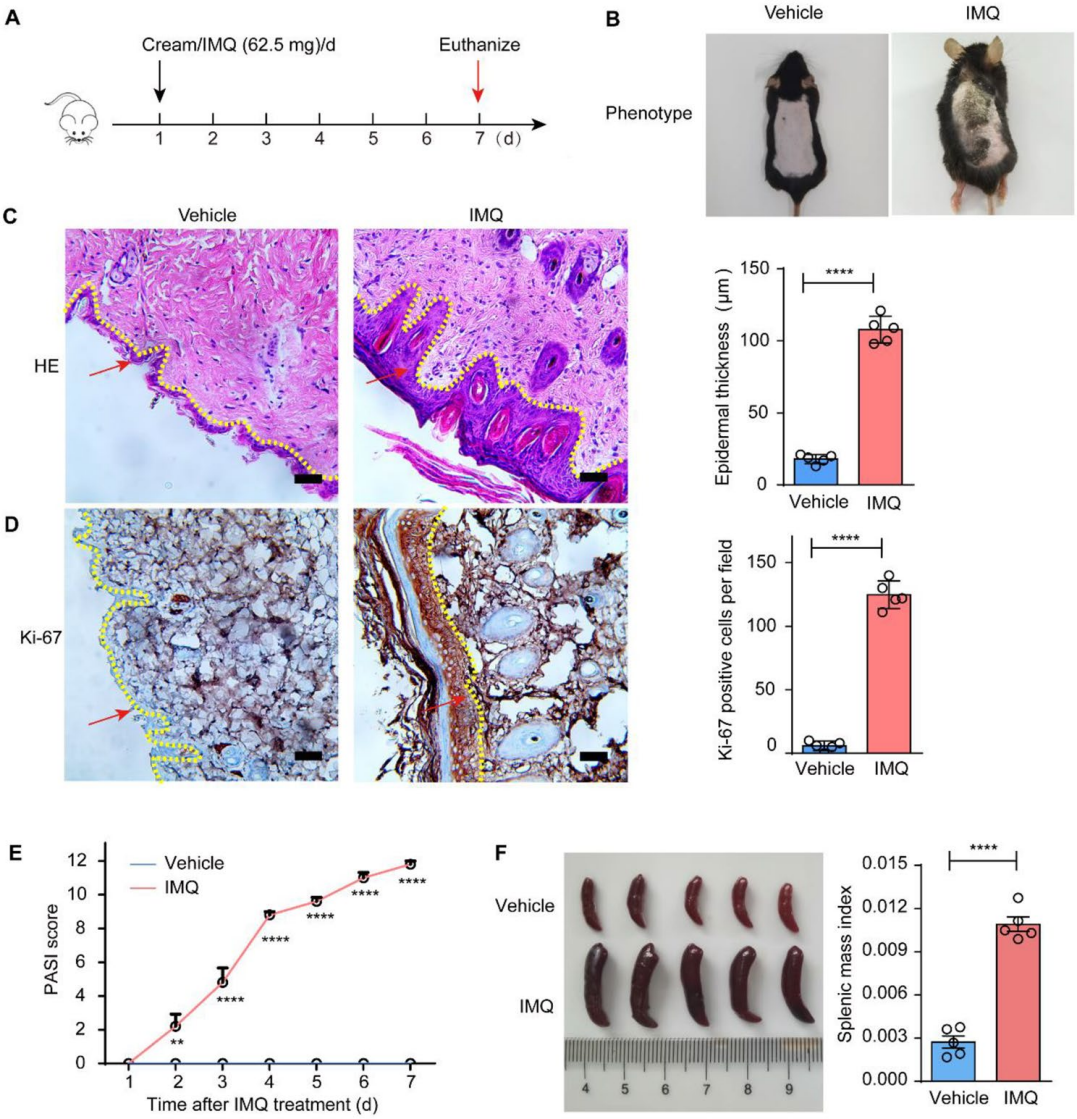
Marked splenomegaly was observed in the psoriatic mice. **A** The setting up of model construction; **B** The phenotype of mice; **C** HE staining of skin in the indicated groups; **D** IHC staining of Ki-67 in the indicated groups; **E** PASI scores after model construction; **F** The spleen phenotype; The splenic mass index (the weight of spleen / the weight of mice)

The psoriasis mouse model was established by subcutaneously administering 62.5 mg of imiquimod (IMQ). The control group received an equivalent amount of Vaseline (Fig. 1A). The mice in the model group exhibited pronounced erythema, scaling and conspicuous skin thickening (Fig. 1B). H&E staining revealed marked epidermal thickening in the model group (Fig. 1C). The proliferative capacity of keratinocytes was significantly increased (Fig. 1D). Prolonged exposure to IMQ exacerbated psoriatic symptoms in mice, as indicated by the Psoriasis Area and Severity Index (PASI), a robust indicator for assessing psoriatic symptoms (Fig. 1E). Splenomegaly is also considered an indicator of worsening symptoms [17]. We observed a significant enlargement of the spleen in the psoriatic mice. The Splenic mass index, defined as the ratio of spleen weight to mouse weight, mitigates the interference of mouse weight on spleen size, thus provides an objective reflection of changes in spleen weight [6]. We found a notable increase in the splenic mass index post-modeling (Fig. 1F). Considering that the spleen is the largest peripheral immune organ in the body [18], we hypothesize that there is a correlation between immune cells within the microenvironment of the spleen and the progression of psoriasis.

### Neutrophils are the main subset that undergoes the most significant changes in the spleen

**Fig. 2:**
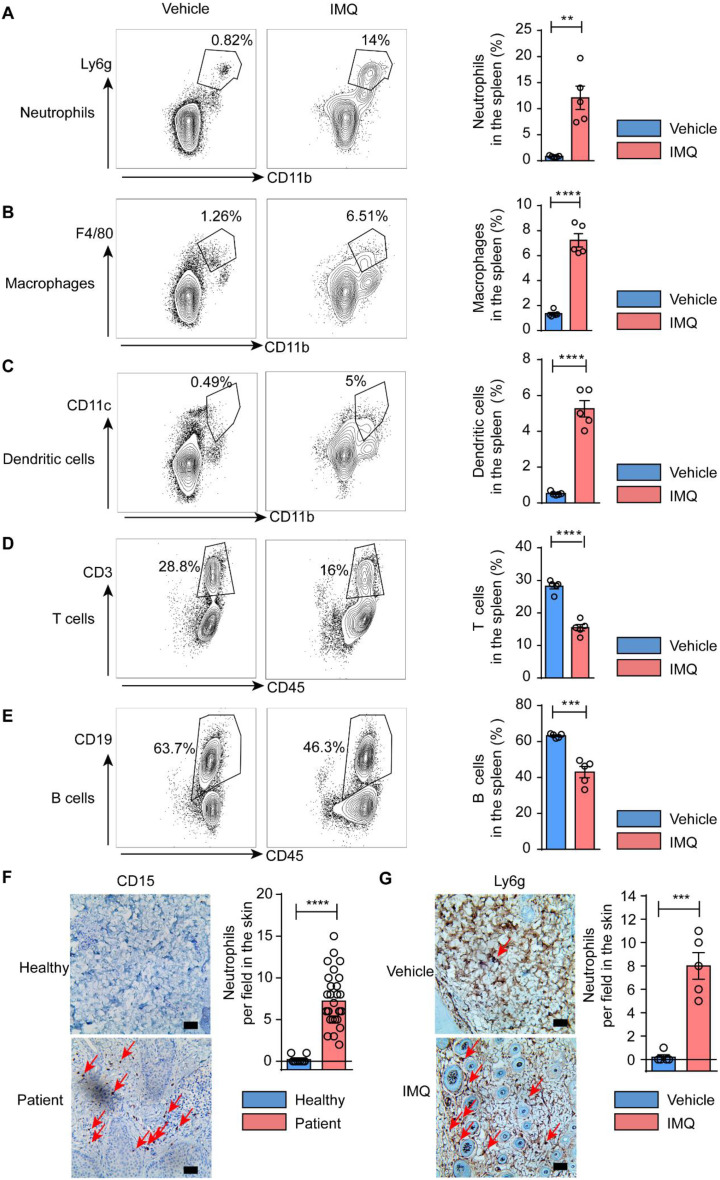
Neutrophils represented the predominantly altered cellular subset in the spleen. **A**-**C** Neutrophils, macrophages and DC cells in spleen; **D**&**E** The percentage of T cells and B cells in the spleen; **F** CD15 expression in healthy and psoriatic patient; **G** Ly6g expression in vehicle and psoriatic model

T cells, B cells, dendritic cells (DCs), and macrophages are also key effectors to the progression of psoriasis. To further investigate the alterations of immune cell populations within the splenic microenvironment of psoriatic mice, we employed flow cytometry to analyze the proportions of these cells in the spleen. Our research findings indicated a substantial increase in myeloid lineage cells, including macrophages, DCs, and neutrophils when compared with the control group (Fig. 2A-C). Conversely, there was a notable decrease in the percentage of T cells and B cells (Fig. 2D-E). Furthermore, neutrophils exhibited the most pronounced changes within the spleen. We also assessed neutrophils in the lesions of both patients and psoriatic mice. A significant neutrophil accumulation was demonstrated in the skin (Fig. 2F and G). These results indicate a potential correlation between neutrophil levels in the spleen and those observed in the skin.

### The depletion of neutrophils improves psoriasis

**Fig. 3:**
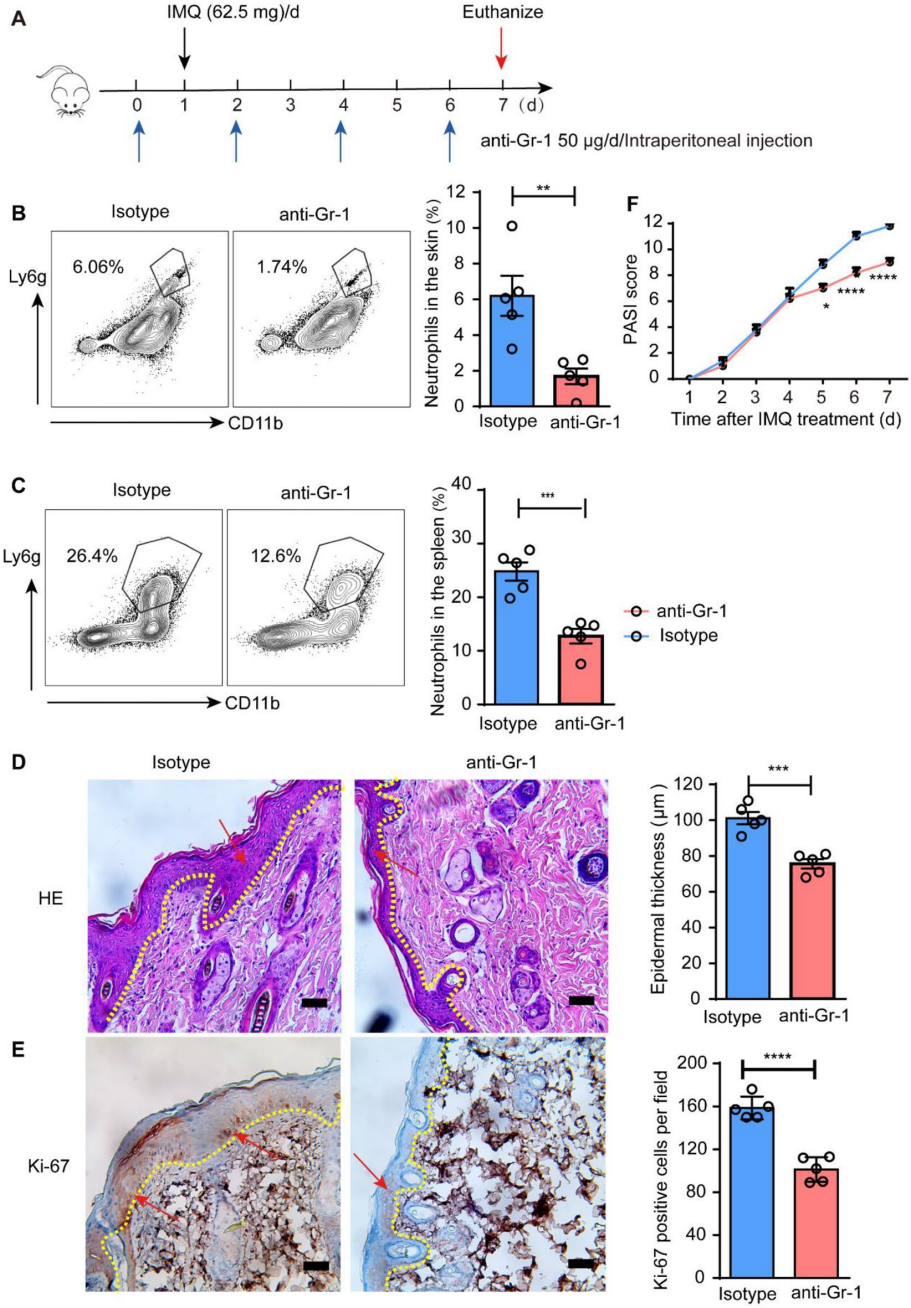
Neutrophil depletion ameliorated psoriatic manifestations. **A** The setting up of model construction; **B**&**C** Neutrophil percentage of spleen and skin was assessed by flow cytometry in the indicated groups; **D** HE staining of skin; **E** IHC staining of Ki-67 in the indicated groups; **F** PASI scores after model construction

Neutrophil accumulation in the skin of psoriatic patients is a significant phenomenon, prompting us to further investigate their role in the progression of psoriasis. To explore this, anti-Gr-1 antibodies were used to deplete neutrophils in psoriasis mice (Fig. 3A). Our findings demonstrated that treatment with anti-Gr-1 resulted in an approximately 90% reduction in neutrophil levels in the skin (Fig. 3B), and about 50% decrease in neutrophils within the spleen (Fig. 3C). The improvement of various symptoms was further demonstrated in the psoriatic mice, including epidermal thinning, reduced ki67-positive cell count, and a significantly decreased PASI scores (Fig. 3D-F). Collectively, these results strongly support the notion that neutrophils play a crucial role in promoting both the onset and progression of psoriasis. Additionally, the levels of spleen neutrophils may be correlated to the severity of psoriasis.

### Splenectomy reduces the infiltration of neutrophils in the skin lesions

**Fig. 4:**
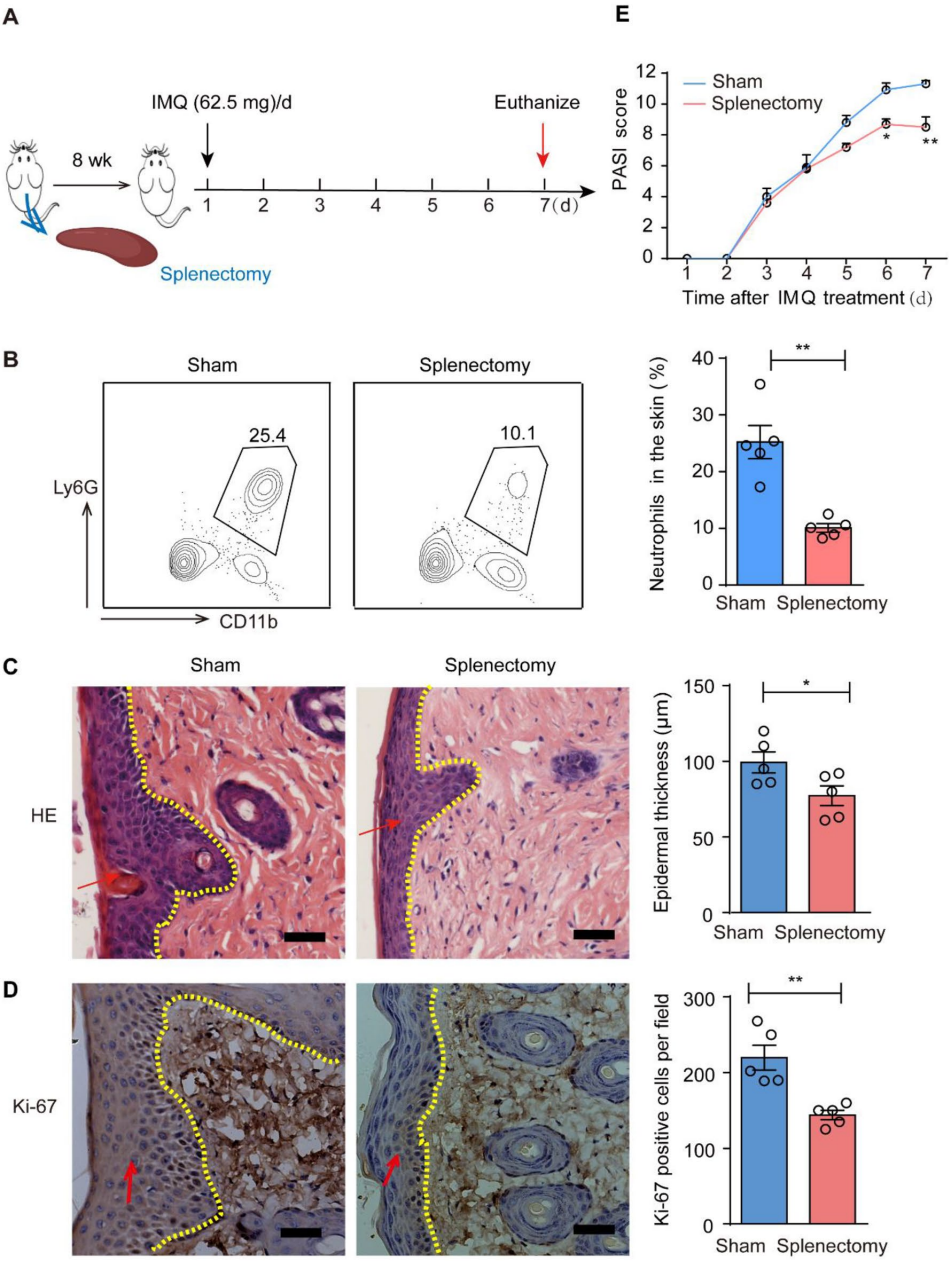
Splenectomy attenuated neutrophil infiltration in psoriatic skin lesions. **A** The setting up of experiment; **B** Neutrophil percentage of skin was assessed by flow cytometry in the indicated groups; **C** HE staining of skin; **D** IHC staining of Ki-67 in the indicated groups; **E** PASI scores after model construction

The spleen has been identified as an extramedullary site responsible for the predominant production of myeloid cells, including neutrophils during the inflammatory response [18]. Thus, we next examined whether the spleen serves as a major source of neutrophils in psoriasis conditions. To address this question, we performed splenectomy on mice and selected mice two months post-surgery to establish our model (Fig. 4 A). As anticipated, neutrophil infiltration in skin lesions of psoriatic mice was decreased after splenectomy (Fig. 4B). Furthermore, our results demonstrated that inflammation was alleviated in mice with splenectomy compared to control mice (Fig. 4 C-E). The above results indicate that reducing the supply of neutrophils through splenectomy may contribute to the reduction of inflammation in the psoriasis-like model. Therefore, spleen may be an important source of neutrophils in the process of psoriasis inflammation.

### The granulopoiesis in the spleen and may be associated with IL-6

**Fig. 5:**
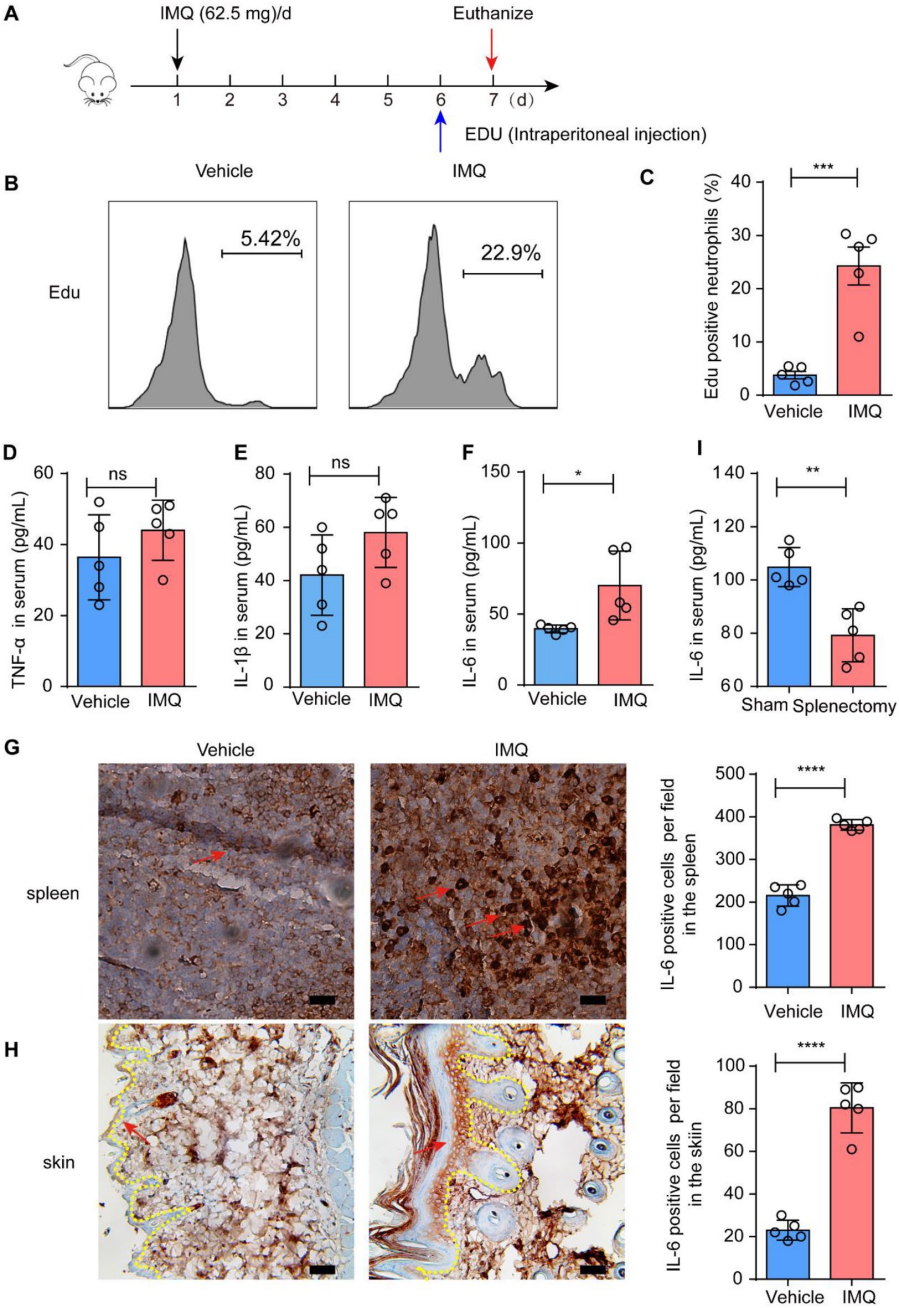
Splenic granulopoiesis appeared to be associated with IL-6 signaling. **A** The setting up of the experiment; **B**&**C** Edu was administered intraperitoneally to examine the formation of neutrophils; **D**-**F** The levels of IL-1β, TNF-α and IL-6 in the serum by ELISA; **G&H **The IHC staining of IL-6 in the spleen and skin; I The levels of IL-6 in the serum of the indicated groups

To further investigate the underlying cause of increased neutrophils in the spleen of psoriatic mice, we administered Edu intraperitoneally to examine the neutrophil formation (Fig. 5 A). The results showed a significant enhancement in Edu signal compared with the control groups (Fig. 5B and C), indicating evident neutrophil formation in vivo. We speculate that inflammatory molecules present in the blood may mediate the neutrophil formation signal within the spleen. Consequently, we proceeded to study changes in inflammatory mediators in the serum. The results indicated no evident changes in IL-1β and TNF-α levels (Fig. 5D and E), while serum IL-6 exhibited a significant increase (Fig. 5 F). Moreover, both spleen and skin showed elevated levels of IL-6 (Fig. 5G-H). It was reported that IL-6 was a key effector related to myelopoiesis [19]. We further examine the changes of IL-6 levels after splenectomy. As we expected, the IL-6 in the serum was significantly decreased after splenectomy (Fig. 5 I). Thus, these results indicate that IL-6 may be responsible for promoting granulopoiesis within the splenic microenvironment of psoriatic mice.

### IL-6 was the key mediator that regulates the neutrophil formation in the spleen of psoriatic mice

**Fig. 6:**
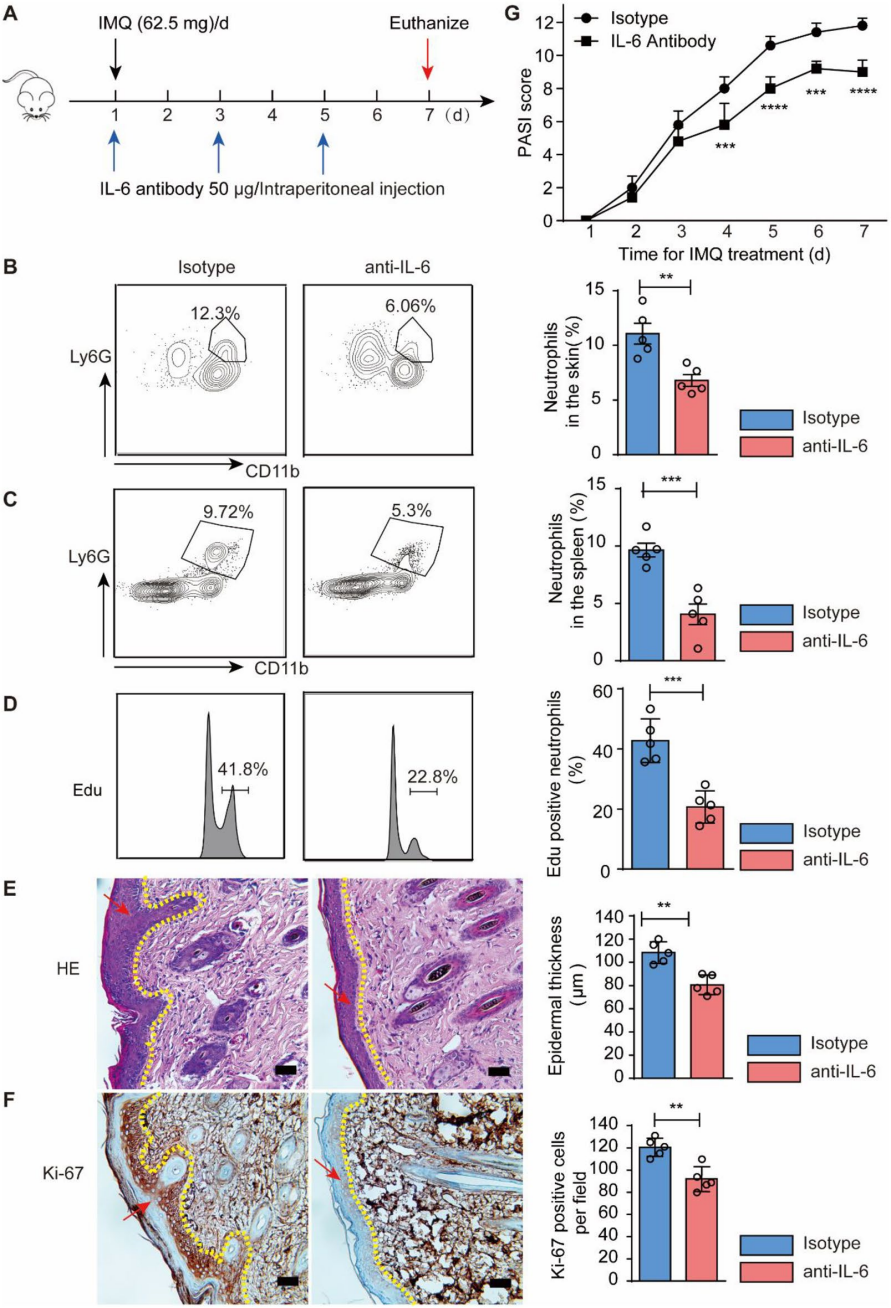
Splenic neutrophil development in psoriatic mice was predominantly regulated by IL-6. **A** The setting up of the experiment; **B**-**C** The neutrophils in the spleen and skin with flow cytometry; **D** The formation of neutrophil was evaluated by Edu injection; **E** Staining of the skin in the indicated groups; **F** IHC staining of ki-67 was evaluated of the skin; **G** PASI scores in the indicated groups

To further investigate that IL-6 is the key mediator responsible for spleen neutrophil formation in psoriatic mice, intraperitoneally administration of IL-6 antibody was performed every other day. Subsequently, on the day 7 of modeling, the mice were euthanized (Fig. 6 A). The results showed a significant decrease in IL-6 levels of spleen and skin following anti-IL-6 treatment compared to the control group (Fig. S1A and B). Notably, skin neutrophils exhibited a marked reduction upon administration of IL-6 antibody (Fig. 6B). Furthermore, there was a significant decrease in spleen neutrophils as well (Fig. 6 C). Additionally, Edu-based measurement revealed lower neutrophil formation compared to the model group (Fig. 6D). Moreover, we observed reduced epidermal thickness and Ki-67 staining intensity in keratinocyte (Fig. 6E and F), along with a decreased PASI score indicating improved psoriasis severity (Fig. 6G). Collectively, these findings support the role of IL-6 as the key regulator of spleen neutrophil formation in psoriatic mice.

### IL-6 may regulate granulopoiesis by P-STAT3

**Fig. 7:**
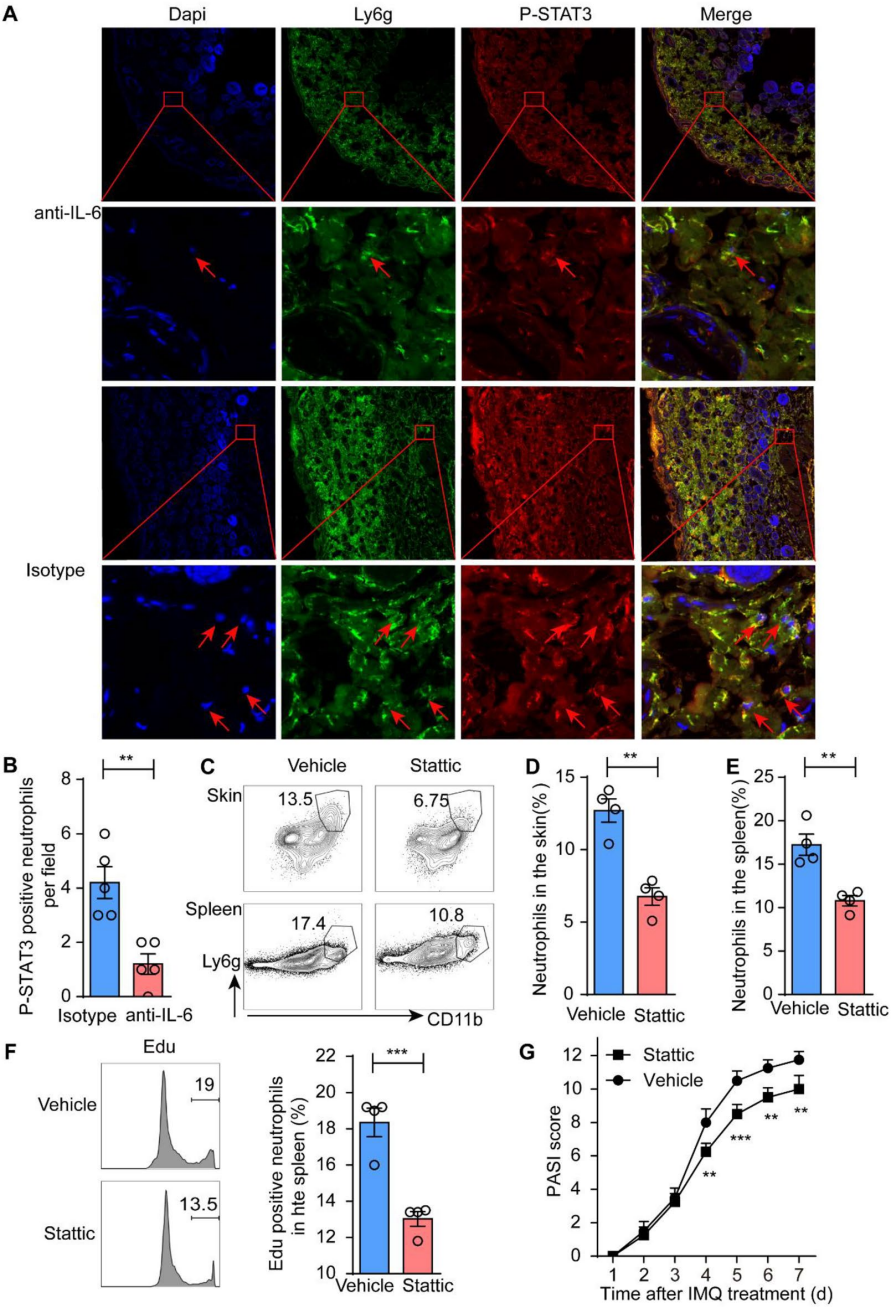
IL-6 may regulate neutrophil formation by P-STAT3. **A**&**B** P-STAT3 in neutrophils were evaluated and the statistical analysis of the staining; **C**-**E** The neutrophil infiltration in the spleen and skin with Stattic treatment and the statistical analysis of neutrophil infiltration; **F** The neutrophil formation in the spleen with Edu tests; **G** PASI scores in the indicated groups

The previous study suggested that P-STAT3 was the key signal way, which was closely associated with hematopoiesis. To further investigate the mechanism by which IL-6 regulate the granulopoiesis, we examine P-STAT3 in the neutrophils. We found P-STAT3 was decreased after IL-6 antibody treatment (Fig. 7 A and B). Thus, IL-6 may regulate granulopoiesis by P-STAT3. To further investigate the role of P-STAT3, we administered the P-STAT3 inhibitor Stattic to psoriasis-like mice. The experimental results demonstrated that the neutrophils in the skin and spleen were significantly reduced (Fig. 7 C-E). The neutrophil formation in spleen was also decreased (Fig. 7 F). CXCL1 is the primary chemokine for skin neutrophils, which was also decreased after Stattic injection (Fig. S1D). Furthermore, the epidermal thickness in the mice was significantly decreased (Fig. S1C), and the PASI scores were improved markedly after the administration of Stattic (Fig. 7G). The results indicated that the neutrophil formation may be regulated by P-STAT3. Therefore, IL-6 regulates neutrophil formation likely through P-STAT3.

## Discussion

The current study provided novel insights into the potential role of the spleen as an extramedullary hematopoietic organ for neutrophils to facilitate neutrophil infiltration into the skin and contribute to the progress of psoriasis. In psoriatic mice, neutrophils were increased in the spleen, while splenectomy inhibited neutrophil infiltration into the skin and alleviated skin inflammation, highlighting the importance of spleen-mediated neutrophil formation. Moreover, IL-6 was identified as a mediator in this process, as anti-IL-6 treatment reduced neutrophil formation by P-STAT3 and subsequently decreased both splenic and cutaneous neutrophil counts, leading to a significant alleviation of inflammation in psoriatic mice. Consequently, this study provides further evidence supporting the clinical application mechanism of IL-6 monoclonal antibodies in the treatment of psoriasis.

### Granulopoiesis, the process of forming granulocytes, primarily occurs in bone marrow

While the process of extramedullary hematopoiesis (EMH), with which blood cells are produced outside the bone marrow, occurs in organs such as the liver, spleen, and lymph nodes [20–22]. EMH can be activated when there is insufficient blood cell production in the bone marrow, due to conditions like atherosclerosis, myelofibrosis, and infections [23, 24]. It has been reported that the spleen, as a lymphoid organ, harbors progenitor cells [14]. In this study, we observed extensive accumulation of neutrophils in the spleen of psoriatic mice, along with significantly increased signals of Edu indicating neutrophil formation. Moreover, splenectomy resulted in a significant reduction in neutrophils within the skin. This phenomenon may be attributed to the supplementary role of the spleen in enhancing bone marrow’s function about neutrophil formation and providing protection. It was also reported that spleen diameter is correlated with the duration of psoriasis [25]. Whether psoriasis patients also exhibit abnormal cell distribution in the spleen requires further experimental verification. Therefore, our findings suggest that the spleen may serve as an extramedullary hematopoietic source for neutrophils in psoriatic mice, and splenic neutrophils contribute to their accumulation within the skin. Our study provides further insights into understanding neutrophils origin in psoriasis.

The administration of IL-6 antibody treatment in a clinical trial involving 264 participants resulted in a significant reduction in the neutrophil-lymphocyte ratio [26], indicating a strong association between IL-6 and neutrophil levels. Similarly, our investigation revealed a substantial elevation of IL-6 in the blood, spleen and skin of psoriasis-like mice, accompanied by an accumulation of neutrophils in the spleen and skin of these mice. Additionally, after injecting IL-6 antibodies, there was a decrease observed in neutrophils within the spleen and skin, accompanied by a reduction of Edu signal for neutrophil formation. It was reported that IL-6 was a key effector related to myelopoiesis [19], suggesting that IL-6 serves as the primary effector for promoting neutrophil formation. Moreover, P-STAT3 was closely associated with granulopoiesis [27], We found P-STAT3 was decreased after treatment with IL-6 antibody. The inhibitor of P-STAT3 reduced the neutrophil infiltration in the spleen and skin, which also decreased the Edu signal for neutrophil formation in spleen. Therefore, the treatment targeting IL-6 could be an effective method to regulate neutrophils and alleviate the inflammation of psoriatic mice. Our study offers a promising therapeutic target by focusing on neutrophils within the psoriatic spleen. However, Further research is warranted to elucidate the precise molecular mechanisms through which IL-6 facilitates neutrophil formation.

Neutrophils are pivotal innate immune cells that play critical roles in inflammation, autoimmune diseases, and cancer [28–30]. Neutrophil infiltration is a primary histopathological hallmark of psoriasis [31]. It has been reported that the progression of psoriasis is predominantly driven by neutrophils through mechanisms involving respiratory burst and granule enzyme release [32]. Our research substantiates this point as we have confirmed significant neutrophil infiltration in both psoriasis-like mice and patients. A previous study indicated that mesenchymal stem cells accelerate peripheral neutrophil clearance, prompting their return to the bone marrow and thereby alleviating corneal injury [33]. Consistent with these findings, we observed effective mitigation of psoriasis symptoms through efficient neutrophil clearance. Consequently, the treatment targeting neutrophils effectively ameliorates the progression of psoriasis.

In summary, our study investigated the origin of neutrophils in the microenvironment of psoriatic mice. We observed a significant neutrophil formation in the spleen, which promoted their infiltration into the skin. Furthermore, we identified IL-6 as a key regulator for neutrophil formation within the spleen, which may be regulated by P-STAT3. The treatment targeting IL-6 or P-STAT3 could effectively inhibit the infiltration of neutrophils in the spleen and skin, thereby alleviating the progression of psoriasis.

## Materials and methods

### Patients and specimens

The patients with plaque psoriasis (12 males and 18 females, aged 22 to 62 years) were recruited from the First Affiliated Hospital of Soochow University. They did not have any concomitant systemic diseases or ongoing treatments. Informed consent was obtained from all participants enrolled in the study. All skin samples were collected following approval by the Ethics Committee of the First Affiliated Hospital of Soochow University.

### Splenectomy

A transverse incision was made below the midline of the left abdominal region to gain access to the surgical site for ligation of the arteries and veins at both ends of the spleen, followed by complete splenectomy. In the sham-operated group, mice underwent a similar abdominal incision and gentle manipulation of the spleen without resection. After surgery, the abdominal cavity was sutured, and mice were monitored until fully conscious. Two months later, IMQ induction was performed to initiate subsequent experimental procedures.

### Animals and IMQ-induced psoriasis model

Male C57BL/6 mice, aged 8–9 weeks, were obtained from Charles River Laboratories (Beijing, China) and housed under specific pathogen-free conditions. In vivo experiments were approved by the Animal Welfare and Utilization Committee of Suzhou University. Initially, the dorsal fur of mice was removed. Then, a daily topical dose of 62.5 mg 5% IMQ cream (Aldara, 3 M Pharmaceuticals, MN) was applied to the dorsum of mice for 6 consecutive days. The Psoriasis Area and Severity Index (PASI) was employed to assess psoriasis symptoms in mice based on erythema intensity, scaling severity, and skin thickness using a scoring scale ranging from 0 to 4: absent (0), slight (1), moderate (2), marked (3), very marked (4).

### Flow cytometry analysis

Single-cell suspensions were prepared from the spleen and skin, and labeled with 7-aminoactinomycin D, anti-mouse CD45 (eBioscience), anti-mouse CD3 (eBioscience), anti-mouse F4/80 (Biolegend), anti-mouse CD11b (eBioscience, CA, ), anti-mouse Ly6g (Biolegend) and anti-mouse CD11c (Biolegend) antibodies. The samples were harvested and analyzed by a Cytoflex Flow Cytometer.

### EdU incorporation

EdU was dissolved in dimethyl sulfoxide and subsequently diluted by phosphate-buffered saline (PBS). This solution was then administered to the mice via intraperitoneal injection 12 h prior to euthanasia. We achieve the cell suspensions of spleen for the neutrophil staining. For the EdU click reaction, the cells were treated with the staining solution, incubated for 20 min in the dark. Then, the cells were washed and resuspend with PBS and analyzed by a Cytoflex Flow Cytometer.

### Infusion of anti-Gr-1 mAb, IL-6 mAb and stattic in vivo

The mice were intraperitoneally administrated with 55 µg anti-mouse anti-Gr-1 (Biolegend) and 60 µg anti-mouse anti-IL-6 (Biolegend), along with an equal amount of IgG (Biolegend) as a control. Additionally, the mice were injected intraperitoneally with 250 µg Stattic (MCE) every other day. On day 7, the mice were sacrificed and both the skin and spleen tissues were collected for Hematoxylin-eosin staining (H&E) or flow cytometry analysis.

### H&E, IHC and IF

The skin and spleen tissues from psoriasis model mice were fixed in a 4% paraformaldehyde solution for 36 h. Subsequently, the tissues underwent dehydration using a series of ethanol concentrations (70%, 80%, 90%, 95%, and finally, 100%). The tissue samples were then treated with xylene and embedded in paraffin. Following this, the samples were sectioned into slices measuring between 3 and 5 μm in thickness. Finally, the tissue sections were stained with hematoxylin and eosin, and subjected to subsequent statistical analysis.

For immunohistochemistry experiments, the tissue sections were deparaffinized and rehydrated prior to undergoing antigen retrieval. Subsequently, the sections were stained with Ki-67 antibody and then incubated with secondary antibodies. Finally, the stained results were analyzed using a microscope.

For IF experiment, the sections were stained with Gr-1 antibody, P-STAT3 antibody, followed by the secondary antibodies. Subsequently, the stained sections were analyzed using fluorescence microscope.

### Statistical analysis

The data were presented as means ± SEM. Statistical analysis was assessed by GraphPad Prism 6 software. Two-sample analysis was conducted using Student’s t-test, while multiple sample analyses were carried out using one-way ANOVA or two-way ANOVA. A significance level of *p* < 0.05 was considered statistically significant.

## Electronic supplementary material

Below is the link to the electronic supplementary material.

Supplementary Material 1

## Data Availability

No datasets were generated or analysed during the current study.
