# Supplementary material for: Spleen granulopoiesis in psoriasis immune microenvironment aggravates psoriasis via IL-6/P-STAT3 signaling
Source: Biol Direct. 2025 Sep 2;20:96. doi: 10.1186/s13062-025-00675-2 (PMC12403492; doi:10.1186/s13062-025-00675-2)
Supplement: Supplementary file 1 — Supplementary Material 1 [file 13062_2025_675_MOESM1_ESM.docx]

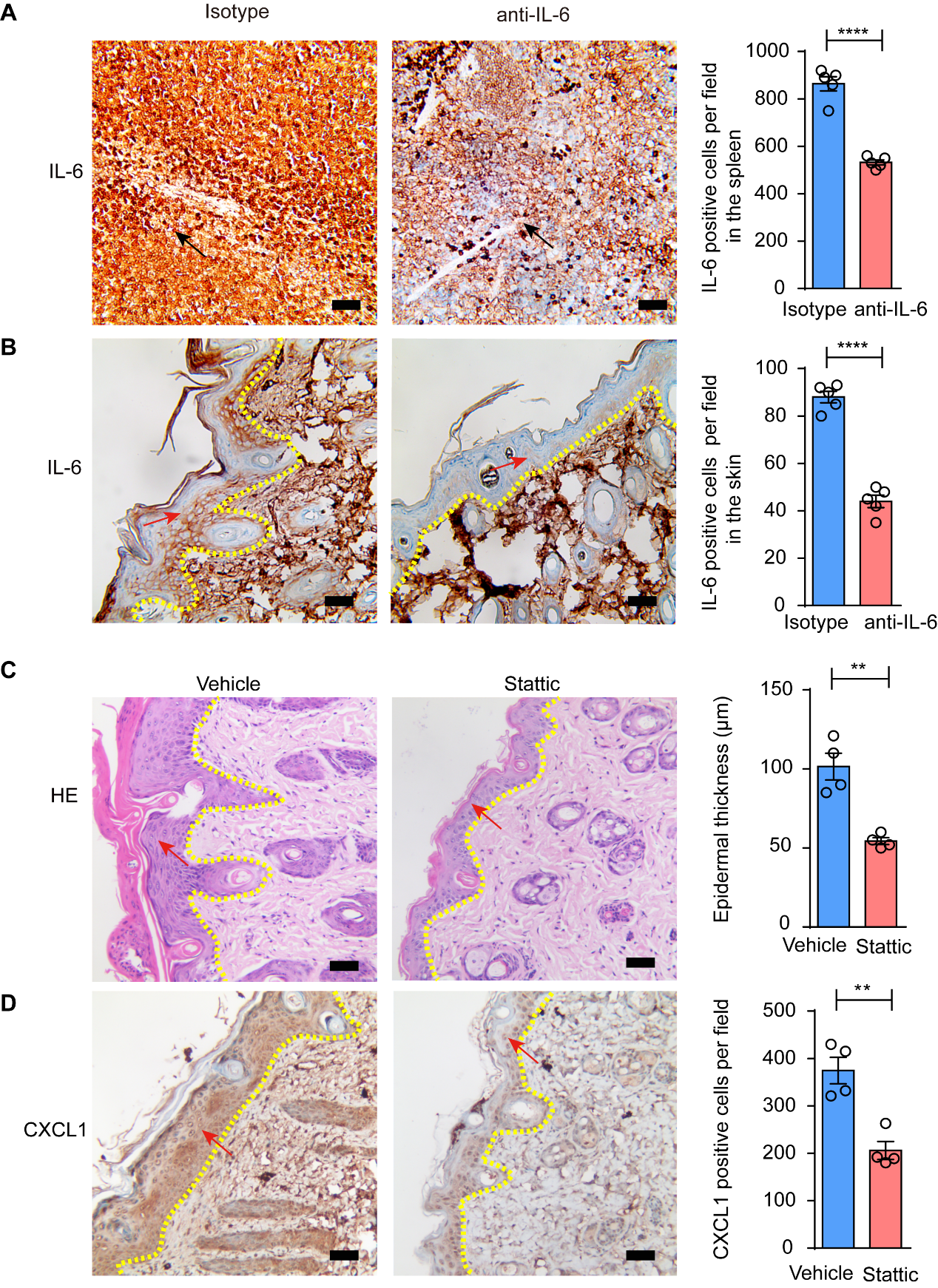


Supplementary 1: A&B IL-6 antibody inhibited the expression of IL-6 in skin and spleen; C Stattic inhibit the epidermal thickness of the psoriatic mice; D CXCL1 expression in the indicated groups of mice with IHC.
